# It’s not who you know: sociometric position did not shape children’s innovation or imitation propensities

**DOI:** 10.3389/fpsyg.2026.1838722

**Published:** 2026-10-16

**Authors:** Bruce Rawlings, Emma Flynn, Rachel Kendal

**Affiliations:** 1 Department of Psychology, Durham University, Durham, United Kingdom; 2 Durham Cultural Evolution Research Centre, Durham University, Durham, United Kingdom; 3 University of Surrey, Guildford, United Kingdom; 4 Department of Anthropology, Durham University, Durham, United Kingdom

**Keywords:** children, classroom, creativity, friendships, imitation, innovation, Sociometric position

## Abstract

**Introduction:**

The capacity for cumulative culture depends on the interplay between social learning and innovation, yet the social-ecological factors that shape individual variation in these processes remain poorly understood. Children’s position within peer networks has been theorised to influence learning strategies by shaping exposure to social models and normative conformity pressures, but this prediction remains untested in experimental contexts.

**Methods:**

We tested whether children’s sociometric position predicted social learning and innovation across three complementary experimental tasks. 256 children aged 7–11 completed sociometric friendship nominations within their school classrooms and three tasks: the Multi-Method Box, a puzzlebox measuring learning strategy choice and innovation-by-modification; a tool innovation task, the hook task; and the Alternate Uses divergent thinking task. We derived three peer-nomination indices — out-degree, reciprocated friendships, and in-degree — and tested their associations with task performance using multilevel models controlling for age and classroom clustering.

**Results:**

Across all tasks and outcome measures, none of the sociometric indices predicted children’s learning strategy choice, imitation fidelity, innovation-by-modification, tool innovation success, solution method, or divergent thinking performance. In contrast, age consistently predicted innovation-related outcomes: older children produced more reward-exit and tool-entrance innovations on the Multi-Method Box, were more likely to solve the hook task and to use a hook rather than a dragging strategy, and generated more divergent thinking responses. These age effects replicated well-established developmental findings and held independently of children’s network position.

**Discussion:**

These findings suggest that individual-level dispositions and developmental cognitive gains may carry greater explanatory weight than relational embeddedness for micro-level learning decisions in middle childhood. Network structure may regulate the flow of cultural information across populations without determining the immediate learning strategy choices of individual children in individually administered experimental contexts.

## Introduction

The ability to build upon, refine, and transmit knowledge across generations is a standout feature of human cognition and culture. Cumulative culture—the process by which innovations accumulate and become increasingly complex over time—depends on the interplay between innovation and social learning. Innovation introduces new ideas, skills, and solutions, while social learning allows individuals to acquire and preserve these advances without rediscovering them from scratch (Whiten, 2019; Legare and Nielsen, 2015; Muthukrishna and Henrich, 2016; Rawlings et al., 2021). The interaction between these two processes enables behavioural flexibility and the ratchet-like accumulation of knowledge that underpins human technological, linguistic, and social complexity. Our capacity for cumulative culture is evident in everything around us, from the multi-step engineering of smartphones and modern transport to the incremental refinement of stone tools and medical technologies, to the scientific methods and institutional norms that allow knowledge to accumulate across centuries (Dean et al., 2014; Derex, 2022; Mesoudi and Thornton, 2018; Rawlings and Reader, 2024).

Developmental research has shown that the foundations of cumulative culture are present in early childhood: young children readily acquire information from others but can also adopt new, more efficient solutions when faced with novel challenges (Davis et al., 2022; Flynn et al., 2016; Flynn and Whiten, 2012; Morgan et al., 2014; Rawlings and Legare, 2021; Subiaul et al., 2015; Whiten et al., 2016). Importantly, they do not learn indiscriminately: children strategically decide when to copy others and when to explore independently, often based on contextual cues, task difficulty, or the perceived competence of others (Carr et al., 2015; DiYanni et al., 2015; Kendal et al., 2018; Morgan et al., 2014; Wood et al., 2013b, 2013a). These learning strategies - the propensity to engage in social learning versus innovation-are thought to help children balance the costs and benefits of imitating versus innovating, ensuring both efficient learning and adaptive flexibility of complex behaviours (Davis et al., 2022; Flynn et al., 2016; Rawlings et al., 2022; Yu and Kushnir, 2020).

## Individual differences in children’s learning strategies

Children vary markedly in whether, and how, they engage in imitation and innovation. Identifying the sources of variation in learning strategies remains critical for understanding how cultural knowledge is acquired and transmitted across development (Flynn et al., 2016; Legare and Harris, 2016; Lew-Levy et al., 2023; Rawlings et al., 2022), as well as how children adapt to new technologies, environments, and challenges. A now relatively large body of experimental work has shown that some children are more likely to imitate observed behaviours faithfully, whereas others prefer to experiment or innovate novel solutions to posed problems (Carr et al., 2015; Flynn et al., 2016; Rawlings et al., 2022; Wood et al., 2013b). Researchers have explored cognitive, cultural, motivational, and dispositional predictors of learning strategies (Beck et al., 2016; Bonawitz et al., 2011; Cutting et al., 2011). For instance, Flynn et al. (2016) showed that children’s learning strategies vary with age and self-evaluation of ability. When 3-5-year-old children were presented with a novel puzzlebox and offered the choice of observing an adult demonstration or attempting to solve it independently, 75% elected for demonstrations, and the older children tended to choose a learning strategy that was effective for them. Using the same paradigm with 7–11-year-olds, Rawlings et al. (2022) examined whether personality was associated with learning strategy choices. Older and conscientious children were more likely to attempt a puzzlebox without observing demonstrations, whereas younger and agreeable children tended to opt for demonstrations. Among those who did observe a model, openness to experience predicted greater deviation from the demonstrated method, indicating a stronger tendency toward innovation (Rawlings et al., 2022).

In addition to explicit learning strategy choices, other work has demonstrated that children differ systematically in how flexibly they balance exploration and exploitation. Bonawitz et al. (2011), for example, showed that children’s exploratory behaviour is sensitive to the informational structure of the environment: when no demonstration was provided on how to use a toy, preschoolers explored objects broadly, but following a clear, intentional demonstration they constrained their exploration to the demonstrated action, highlighting how uncertainty and instructional context jointly shape innovation. Wood et al. (2013a, 2013b) found that children selectively copy efficient actions to solve a puzzlebox, while omitting causally irrelevant steps, with substantial individual variation in fidelity depending on task demands and prior experience. Work on tool innovation has further shown that success is associated with the propensity to try different methods (Evans et al., 2021; Guo et al., 2025), engage in metacognitive and cognitive private speech (Breyel and Pauen, 2023), and to solve other novel problems individually instead of observing demonstrations first (Rawlings et al., 2022).

These findings indicate that children’s learning strategies emerge from an interaction of cognitive development and individual disposition. However, these influences capture only part of the broader social ecology in which learning occurs. Much of what we know about children’s learning strategies comes from dyadic paradigms in which a single demonstrator models behaviour to an individual child—a setting that captures relatively little of the social complexity children encounter in everyday peer contexts. When children observe multiple demonstrators, as they routinely do in naturalistic settings, their imitation becomes markedly more selective: Evans et al. (2018) showed that when a majority demonstrated a causally irrelevant action, children did not blindly copy but calibrated their imitation to the efficiency of what the majority modelled — evidence that children integrate social frequency information in sophisticated, adaptive ways. This suggests that children’s learning strategies are sensitive to the social composition of the learning environment, not just its informational content. A critical next step, therefore, is to examine how the broader structure of children’s everyday social environments—particularly their friendship networks—might shape their opportunities for, and engagement in, social learning and innovation.

## Peer learning as a context for cultural transmission

Children’s friendships are among the most influential contexts for learning, development and socialisation. Across cultures, much everyday learning occurs within peer groups as well as within adult–child dyads (Collier, 1980; Flynn and Whiten, 2012; Lancy, 2010; Lew-Levy et al., 2017; Lew-Levy et al., 2023; Whiten and Flynn, 2010). Childhood peers serve as powerful models for imitation, particularly in instrumental and play contexts where children attend closely to the actions of similar-aged others (Flynn and Whiten, 2012; Lew-Levy et al., 2023; McGuigan and Robertson, 2015; Zmyj and Seehagen, 2013). Peers also act as important sources of novel information, contributing to the generation, refinement, and spread of behavioural variants through interaction, collaboration, and informal teaching (Lew-Levy et al., 2023; Rogoff et al., 2003). In addition, peer interaction provides a social context in which norms are established and maintained through reinforcement, comparison, and coordination, while supporting broader cognitive and social skills such as perspective-taking, problem-solving, and collaborative reasoning (Bukowski and Cillessen, 1998; Rubin et al., 2007; Stengelin et al., 2020). From a cultural-evolutionary perspective, peer interaction is thought to play a key role in sustaining behavioural diversity, facilitating horizontal transmission and enabling the diffusion and modification of innovations within populations, particularly during childhood when peer relationships are frequent and socially salient (Lew-Levy et al., 2023).

However, peer influence is unlikely to be uniform. Children differ markedly in how embedded they are within their peer groups and in the structure of the friendships they maintain (Bukowski and Cillessen, 1998; Gest et al., 2001; Newcomb et al., 1993; Rawlings, 2018; Rawlings et al., 2017; Rubin et al., 2007). In children who attend school, classroom friendship networks also vary in size, reciprocity, and stability, and these differences may shape children’s access to social information and opportunities for learning (Gest, 2006; Gest et al., 2003; Rawlings et al., 2017). From a developmental perspective, children with larger or more reciprocated friendship networks may be exposed to a greater diversity of models, strategies, and perspectives, increasing opportunities for observational learning and social transmission (Kratzer and Lettl, 2008; Rawlings et al., 2017). Gradassi et al. (2023) showed that adolescents’ use of social information decreased substantially with network distance from the demonstrator, an effect fully mediated by subjective closeness to that peer. They also found that demonstrators occupying more central network positions exerted greater influence on learners’ decisions than more peripheral ones. Notably, however, this evidence concerns learning directed at specific, identifiable peers who differ in how close they are to the learner. Thus, it reflects a relational effect, in which children learn most from the particular friends they feel closest to, rather than a more general tendency to rely on social information that a child would carry into any learning encounter. More central children may also experience stronger normative pressures to conform to shared practices, potentially favouring imitation over exploration (Gradassi et al., 2023; Over and Carpenter, 2012; Pinho et al., 2021). From this perspective, individual differences in network position could plausibly relate to children’s learning strategies, though the expected direction is not straightforward. Evidence that more central children exert greater influence as models (Gradassi et al., 2023) concerns their role as sources of information rather than as learners, and does not by itself specify whether central children should themselves copy more or innovate more. A further possibility is that it is children connected to central peers, rather than central children per se, who rely most on social information. We therefore treated the association between embeddedness and learning strategy as an open question and, because our paradigm indexes a child’s general orientation toward social information rather than learning from specific peers, focused on whether embeddedness relates to the social-versus-asocial decision at all rather than committing to a strong directional account.

However, direct tests of how children’s friendship networks relate to their learning strategy choices in problem-solving contexts remain rare. To our knowledge, no developmental study has explicitly examined whether sociometric properties are associated with children’s propensity to engage in innovation or imitation. Insight can, however, be drawn from related lines of research. Gradassi et al. (2023) combined social network analysis with an experimental social learning task in high school classrooms, showing that pupils were more likely to use information from peers who were closer to them in the friendship network. Importantly, this effect was driven by how close pupils felt to those peers rather than by network position alone, with more central and academically respected peers also exerting greater independent influence on learning. Similarly, van Rijsewijk et al. (2016) showed that adolescent friendship relations, measured via peer nominations, influenced prosocial behaviour: adolescents were more likely to help friends, reciprocate help within mutual friendships, and provide direct assistance to peers with higher social status in the social network. Network-based studies of trust and cooperation demonstrate that children preferentially direct cooperative and helping behaviours toward friends and socially central peers, with reciprocity and popularity shaping who is considered a reliable interaction partner (Balliet et al., 2014). Adult research on creativity and social influence in the workplace suggests that social embeddedness can both enable and constrain exploratory behaviour: individuals who are highly embedded in social networks often conform more closely to group norms, whereas less-connected or peripheral individuals may be more willing to deviate and generate novel ideas (Baer, 2010; Baer et al., 2015).

Together, these findings suggest that friendship networks play a powerful role in structuring who interacts with whom and how social behaviour is distributed. Much of this evidence, however, concerns behaviour directed toward, or information acquired from, particular familiar peers, and thus bears most directly on a relational account in which embeddedness shapes learning from specific known models. A distinct possibility is that embeddedness instead reflects a more general orientation toward social information — a disposition to rely on, or deviate from, social models irrespective of who provides them. These accounts leave open a key question: whether children’s position within peer networks shapes their propensity to rely on social information or to innovate when solving novel problems.

## The present study

The current study extends Rawlings et al. (2022) by examining whether children’s sociometric position predicts social learning and innovation across multiple tasks. Using classroom friendship nominations in a sample of UK 7-11-year-olds, we calculated how many peers each child nominated, how often they were nominated by peers, and how many friendships were reciprocated, and tested whether these sociometric indices were associated with children’s explicit learning strategy choices and innovation behaviours across a puzzlebox task, a tool innovation and a divergent thinking task. The central aim was to test whether children’s sociometric position predicted their learning strategies, imitation fidelity, tool innovation, and creativity. Because the social option was a single, standardised model whose competence and relevance were held constant across participants, this approach tests whether embeddedness shapes children’s general orientation toward social information, rather than the distinct, relational possibility that it governs learning from specific familiar peers (a question we return to in the Discussion).

Based on cultural evolutionary theory and developmental research on peer learning, we made three predictions. First, regarding learning strategy choice, we predicted that children with greater network embeddedness — indexed by more friends nominated, more reciprocated friendships, and higher in-degree (nominated by peers) — would show a stronger general orientation toward social information, and thus be more likely to explicitly elect for a demonstration on a multi-method puzzlebox than those with lower network embeddedness (Haun and Tomasello, 2011; Over and Carpenter, 2012; Rawlings et al., 2017). Second, given theoretical accounts linking network centrality with conformity to shared practices (Boyd and Richerson, 1985; Kratzer and Lettl, 2008; Morgan et al., 2012), we predicted that, among children who elected for demonstrations, more socially embedded children would show higher imitation scores than less socially embedded children. Third, in line with adult network research indicating peripheral individuals are more willing to generate novel ideas (Baer, 2010; Baer et al., 2015), we predicted that children with lower network embeddedness would show greater innovation-by-modification (modifying observed behaviours) and higher performance on the innovation (hook) and divergent thinking task. Consistent with prior work, we also expected age to positively predict tool innovation, hook task success, and divergent thinking fluency, independently of network position (Carr et al., 2015; Colbourne et al., 2024; Rawlings et al., 2025; Rawlings and Legare, 2021).

## Methods

### Participants and design

This study directly extended Rawlings et al. (2022), using a subset of the same child participants (which was *N* = 282 in that study), but replaced personality measures with sociometric indices of children’s peer relationships. Here, participants were 256 children (131 girls; age range = 7.10–11.90 years, M = 9.34, SD = 1.19) recruited from 22 classrooms across 10 state (non-fee-paying) primary schools mostly in Northeast England (M = 11.6 participants per classroom, SD = 6.3). Although individual ethnicity data were not collected, the ethnic group breakdown of the local area is 96% white, 2.3% Asian/British Asian, 1.1% mixed ethnic groups, 0.3% black/black British, and 0.4% other. Classrooms served as naturally occurring peer groups, and children were tested individually during the school day. Parental consent and child assent were obtained prior to participation. Children were given stickers and stationery gifts as rewards for participation. Ethical approval was granted by Durham University’s Department of Anthropology Ethics Committee.

### Sociometric friendship measures

Children completed a standard peer-nomination task within their school classroom (Freire et al., 2024; Gest, 2006; Gest et al., 2003; Neal, 2011, 2025), in which they were asked to name up to five close friends in their class. We limited the number to five because this cap is widely used in classroom-based sociometric research as a pragmatic compromise that captures children’s core friendship ties while reducing respondent burden and recall error, and it aligns very closely with empirical estimates of the average number of close friends children typically nominate in school settings (Neal, 2025). Capped nomination procedures are intended to capture children’s core, most salient friendships. These are the ties most likely to carry social influence, rather than an exhaustive census of every acquaintance, and, as noted, this closely matches the number of close friends children nominate on average at this age (Neal, 2025). Capped procedures require children to be selective and are therefore well suited to identifying a child’s closest friendships rather than broader peer acceptance (Gommans and Cillessen, 2015), which is the construct of theoretical interest here. Further, and crucially, research shows that unlimited and limited nominations produce comparable results (Gommans and Cillessen, 2015). We also note that this constraint bears principally on a single index. In-degree, a direct measure of a child’s centrality within the classroom peer group (Cillessen and Marks, 2017), is determined by how many classmates nominate a child rather than by that child’s own nomination limit, and captured substantial variation across its full range in the present sample (0–10; Table 1).

**Table 1:** Descriptives of sociometric indices.

Sociometric index	Mean (SD)	Range
Out-degree (no. friends named)	4.35 (1.33)	0–5
In-degree (no. times named by peers)	1.76 (1.79)	0–10
Reciprocated friends (no. mutual friendships)	0.84 (1.03)	0–4

For each child we derived three established developmental network measures:

- Number of friends nominated (out-degree): count of peers each child listed (0–5).
- Reciprocal friendships: count of nominations where both children named each other.
- Times nominated by peers (in-degree): the number of classmates who named the child.

As is common in school-based sociometric research, not all nominated peers participated in the study due to caregiver consent constraints (Cillessen, 2009; Gest et al., 2001; Huisman and Steglich, 2008). However, children were free to nominate any classmate regardless of participation status, meaning that outgoing nominations (number of friends named) reflect children’s perceived social relationships rather than the study sample. Because reciprocity and nominations received can only be computed among participating children, these indices may be attenuated by incomplete participation; however, this limitation is typical of classroom sociometric work (Cillessen and Marks, 2017; Neal, 2020; Veenstra et al., 2013). Moreover, our primary interest was in children’s local embeddedness within the classroom social ecology, rather than reconstructing complete classroom networks - an approach that has been used in developmental network research and is appropriate for testing associations between individual network position and behavioural outcomes (Gest et al., 2011; Laursen and Veenstra, 2021; Neal, 2020, 2025)

### Multi-method box (MMB)

*Administration*: The MMB is a puzzle apparatus containing capsules with sticker rewards (Carr et al., 2015; Rawlings et al., 2022). It includes three tools, multiple possible tool entrances, and multiple reward (capsule containing sticker) exits (Figure 1). As this was part of the same study as Rawlings et al. (2022), the methodology was identical. The experimenter first explained that the goal was to get the sticker capsules out of the box, and then children were asked: “Would you like to have a go yourself or watch me have a go first.” To reduce any potential learning bias due to perceived demonstrator expertise, it was explained that the MMB belonged to a friend (Carr et al., 2015; Rawlings et al., 2022).

**Figure 1:**
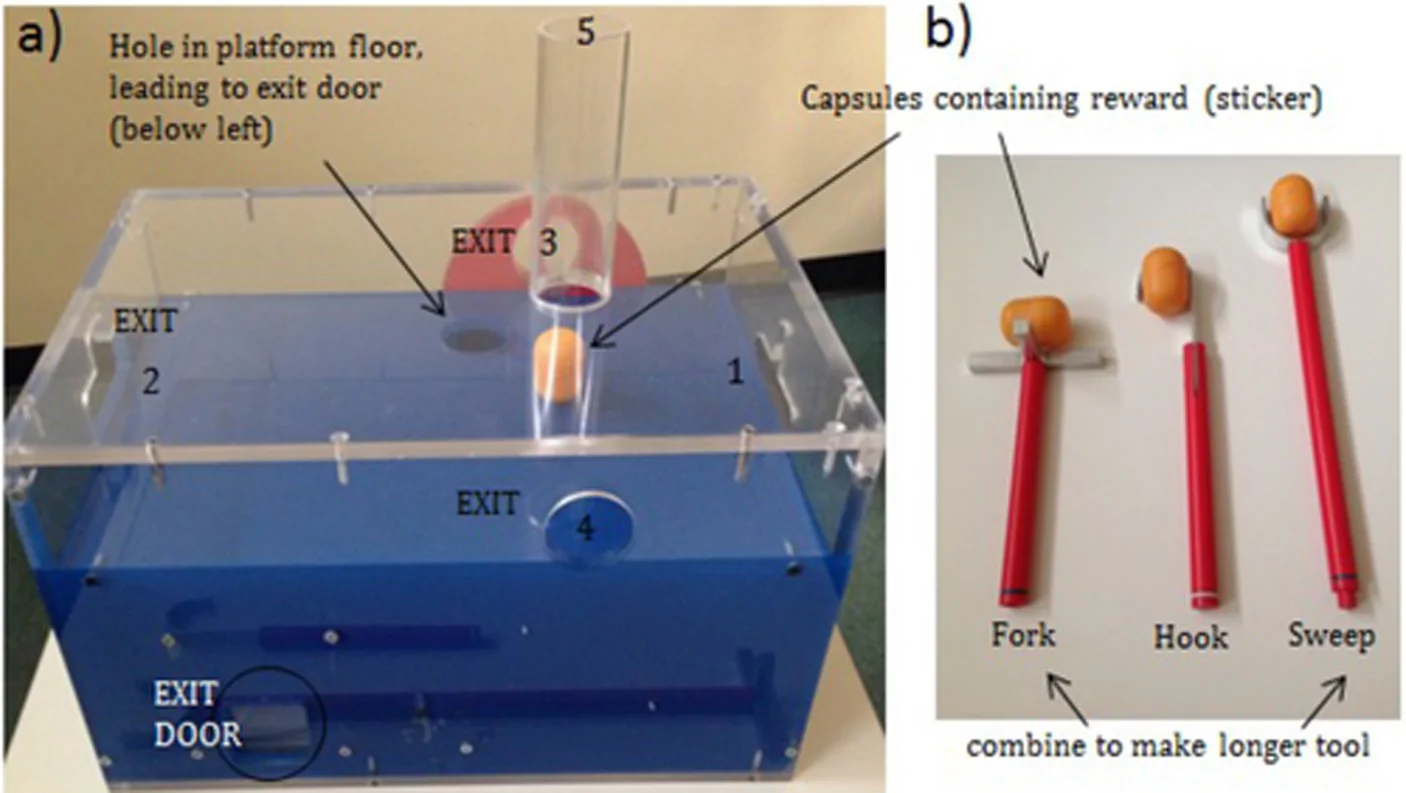
The multi-method box and associated tools. **(a)** the Multi-Method Box apparatus, including the entrances/exits and reward capsule; **(b)** the tools used with the Multi-Method Box task.

Children who elected to observe the experimenter received four demonstrations: three unsuccessful attempts and one successful demonstration using the fork tool via a specific entrance–exit combination. Children who chose to go it alone (asocial learning) received no demonstrations. All children then completed 10 attempts to extract rewards independently.

*Coded measures*: As with Rawlings et al. (2022) and Carr et al. (2015), we coded the following MMB behaviours across all 10 attempts:

### All participants

- Whether elected for demonstrations (coded 0), or no demonstrations (coded 1).
- The number of failed attempts (max. 10).
- The number of different tools used across all attempts (max. 4).
- The number of different entrances used across all attempts (max. 5).
- The number of different exits used across all attempts (max. 6).

### Children who elected for demonstrations only

- The number of imitations across all attempts; matching one or more of the same tool, entrance, and exit as the demonstration (max. 30).
- The number of exit innovations across all attempts; where a different exit than the demonstration was used (max. 10).
- The number of tool innovations across all attempts; where a different tool than the demonstration was used (max. 10).
- The number of entrance innovations across all attempts; where a different entrance than the demonstration was used (max. 10).

### Children who did not elect for demonstrations only

- The number of different tools used across all attempts.
- The number of different entrances used across all attempts.
- The number of different exits used across all attempts.
- The number of repetitions; the total number of times, across all attempts, that an individual repeated a previously used method (of tool, entrance, and exit) exactly (max. 9).

### Hook task

*Administration*: The hook task is a well-established tool innovation challenge known to be difficult for children, particularly in early and middle childhood (Beck et al., 2011; Colbourne et al., 2024; Rawlings, 2022). Children were shown a transparent vertical tube housing a small bucket containing a sticker reward beyond direct reach. They were provided with a straight pipe cleaner and a 15-cm length of string, the latter serving as a distractor item. To retrieve the reward, children needed to modify the pipe cleaner into a hook shape and use it to lift the bucket by its handle out of the tube (Figure 2).

**Figure 2:**
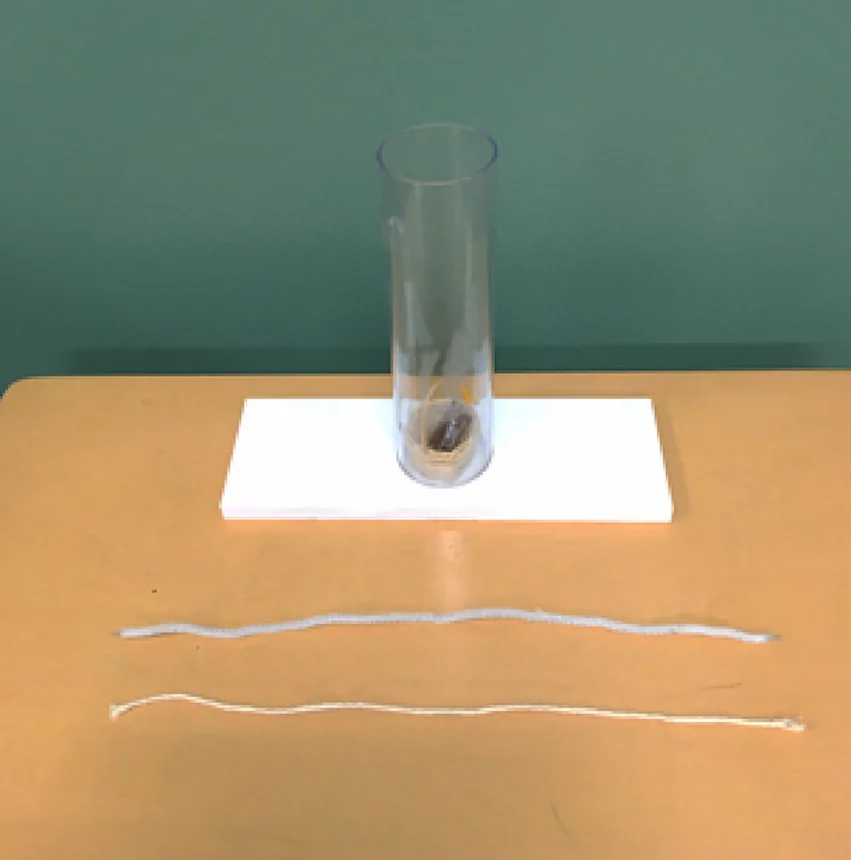
The hook task.

At the start of the task, the experimenter drew the child’s attention to the apparatus and said, “Can you see the sticker inside this tube? See if you can get it out.” No additional guidance or feedback was given during testing. Children were allowed up to 3 min to attempt the task (Rawlings et al., 2022).

*Coded measures*: Successful retrieval of the sticker was scored as 1, and unsuccessful attempts as 0. For children who succeeded, the latency to retrieve the reward was recorded in seconds. In line with Rawlings et al. (2022), we also recorded the method use for success. All children received a sticker at the end of the task regardless of the outcome.

### Divergent thinking

*Administration*: Divergent thinking was assessed using an Alternate Uses task (Guilford, 1967), in which children were asked to generate multiple possible uses for a familiar object. Each child was presented with a paperclip, and the experimenter said, “Here is a paperclip. Tell me all the different things a paperclip could be used for — all the things you could do with it.” As is standard in divergent thinking assessments, children were not explicitly instructed to be creative, to avoid constraining responses or introducing demand characteristics (Rawlings et al., 2025; Runco et al., 2005). To support creative performance, no time limit was imposed (Said-Metwaly et al., 2020). The task continued until the child indicated that they could not think of any further uses and confirmed that they were finished. Only responses that clearly described a functional use were scored; vague answers (e.g., “bending”) were excluded unless accompanied by a specific outcome or purpose (e.g., “bending it into a hook”).

*Coded measures*: Two outcome measures were derived. Fluency was calculated as the total number of distinct, scorable uses generated. Originality captured the relative rarity of responses within the sample: uses mentioned by 2–5% of participants were awarded one point, and uses mentioned by 1% or fewer participants were awarded two points. Responses produced by more than 5% of participants were scored as zero for originality (Rawlings et al., 2025; Said-Metwaly et al., 2020).

### Statistical approach

We tested whether children’s sociometric indices predicted social learning and innovation outcomes using mixed-effects models that accounted for clustering by classroom. In all models, the focal predictors were three peer-nomination indices—number of friends nominated (out-degree), number of reciprocal friendships, and number of times nominated by peers (in-degree)—entered simultaneously alongside age as a covariate. Prior to fitting models, we assessed multicollinearity among predictors using variance inflation factors (VIFs). All VIFs were acceptably low (out-degree: VIF = 1.12; reciprocated friendships: VIF = 2.51; in-degree: VIF = 2.49; age: VIF = 1.06), indicating no problematic collinearity. Classroom (defined as school × year group) was modelled as a random intercept to account for non-independence among children within the same classroom.

For the MMB task, children’s learning strategy choice (asocial versus observing demonstrations) was analysed using a binomial mixed-effects logistic regression. The classroom random effect variance was negligible for this model (variance ≈ 0, singular fit); a sensitivity analysis using a standard logistic regression (without classroom random effect) produced identical parameter estimates. Behavioural outcomes recorded as counts—number of failed attempts, imitation scores, and innovation counts—were analysed using negative binomial mixed-effects models (NB2) fitted with the glmmTMB package. Outcomes expected to contain excess zeros based on prior inspection—specifically, the number of different tools, entrances, and exits used (across both all-participant and asocial subsets), and tool innovations (demonstration subset)—were analysed using zero-inflated negative binomial mixed-effects models (ZI-NB2). Observation-level random effects (OLREs) were included as a precautionary measure in all count models to accommodate any residual overdispersion beyond that absorbed by the negative binomial dispersion parameter.

For the hook task, success (0/1) was analysed using a binomial mixed-effects logistic regression. Among children who succeeded, latency to retrieval (seconds) was analysed using a linear mixed-effects model. Solution method was categorised as hook (pipe cleaner bent into a hook), drag (dragging strategy without hook modification), or other (other idiosyncratic techniques such as making the pipecleaner into a pincer shape)—with analyses restricted to successful trials (*N* = 210). Solution method was analysed using multinomial logistic regression with drag as the reference category. The multinomial model does not include a classroom random effect, as the nnet:multinom function does not support random effects. Finally, divergent thinking outcomes from the Alternate Uses task (fluency and originality) were analysed using linear mixed-effects with Satterthwaite approximation for denominator degrees of freedom.

Model fit was assessed using the DHARMa package for all GLMMs and glmmTMB models, and by inspection of residual-versus-fitted and Q–Q plots for linear mixed models. DHARMa diagnostics indicated residual overdispersion in six count models: number of tools, entrances, and exits in both the all-participant and asocial subsets. Results from these models should therefore be interpreted with appropriate caution. The ZI-NB2 model for asocial tool use additionally produced a non-positive-definite Hessian at convergence, suggesting potential over-parameterisation in this subsample (*N* = 93), so caution is also required when interpreting findings. All remaining count models—failed attempts, imitation, exit innovations, entrance innovations, and asocial repetitions—and the hook-success model passed the relevant DHARMa diagnostic checks. Visual residual diagnostics were acceptable for hook-task latency and AU fluency, whereas AU originality showed a substantial floor effect and was therefore interpreted cautiously. The multinomial hook-method model was not assessed using DHARMa.

To assess whether the null sociometric associations were robust to classroom size and participation rate, we re-fitted all models with in-degree and reciprocity standardised within classroom. Participation rates varied across classrooms, motivating these additional proportional and within-classroom standardisation analyses. Indices were expressed as a proportion of the participating classmates available to nominate each child (received or reciprocated nominations divided by the number of participating classmates minus one) and as within-classroom *z*-scores. Out-degree, in-degree, and reciprocity as originally coded returned no associations with any outcome; the standardised versions produced only isolated coefficients reaching *p* < 0.05, but these were not replicated across the two standardisation schemes for any single index, consistent with chance rather than a systematic network position effect.

## Results

We examined whether peer network scores predicted learning strategy choice, social learning, innovation, and creativity across three tasks. Table 1 displays descriptive data for the sociometric indices.

### Learning strategy choice on the multi-method box (MMB)

As this sample comprised a subset of the 282 children reported in Rawlings et al. (2022), children’s learning strategy choices closely mirrored those observed in the full dataset. Overall, 63.7% of children (*N* = 163) elected to observe a social demonstration before attempting the MMB, whereas 36.3% (*N* = 93) chose to attempt the task asocially without demonstrations. Girls were more likely to elect for demonstrations than go it alone (73.8%; 96 of 130), whereas boys were more evenly split (53.2% choosing demonstrations; 67 of 126).

A binomial mixed-effects logistic regression revealed no evidence that any sociometric measure predicted learning strategy choice (out-degree: *β* = −0.09, SE = 0.10, *z* = −0.84, *p* = 0.400; reciprocity: *β* = 0.04, SE = 0.21, *z* = 0.19, *p* = 0.853; in-degree: *β* = −0.06, SE = 0.12, *z* = −0.48, *p* = 0.629). Age was also not a significant predictor (*β* = 0.20, SE = 0.11, *z* = 1.76, *p* = 0.079). Children who nominated more friends, had more reciprocated friendships, or were nominated more frequently by peers were no more or less likely to request demonstrations than their peers. The classroom random effect variance was estimated at zero, indicating negligible between-classroom variation in strategy choice; a sensitivity analysis using a standard logistic regression (no random effects) produced identical parameter estimates. For all model effects, see Figure 3.

**Figure 3:**
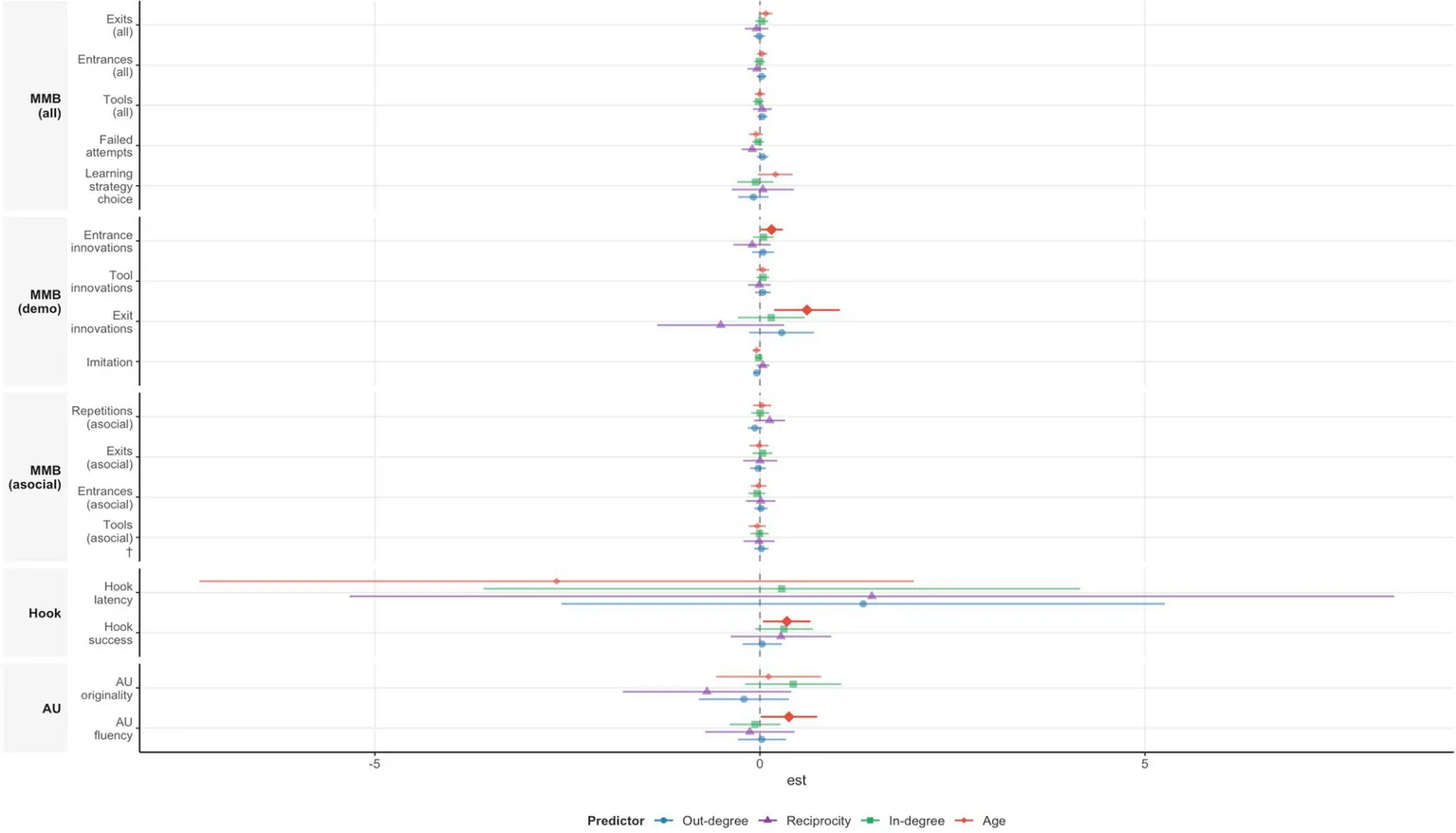
Sociometric predictors and age effects across all outcomes.

### MMB performance across all participants

We examined whether sociometric indices predicted overall MMB engagement across all children, irrespective of prior learning strategy choice. The mean number of failed attempts was 3.21 (SD = 2.17). None of the sociometric predictors were associated with failed attempts (out-degree: *β* = 0.04, SE = 0.04, *z* = 0.93, *p* = 0.345; reciprocity: *β* = −0.10, SE = 0.07, *z* = −1.45, *p* = 0.150; in-degree: *β* = −0.02, SE = 0.04, *z* = −0.62, *p* = 0.545), and age was also not a significant predictor (*β* = −0.05, SE = 0.04, *z* = −1.11, *p* = 0.262).

We then analysed children’s breadth of exploration, indexed by the number of different tools (M = 2.40, SD = 0.95), entrances (M = 2.43, SD = 1.09), and exits (M = 1.61, SD = 0.87) used across all attempts. ZI-NB2 models with OLREs were fitted for all three outcomes. DHARMa diagnostics flagged residual overdispersion in all three models (all *p*s < 0.001), results should therefore be interpreted with caution. Across all three measures, there was no evidence that sociometric indices predicted exploration breadth (all *p*s ≥ 0.340). Age was also not a significant predictor of the number of different tools (*β* = −0.001, *p* = 0.983), entrances (*β* = 0.03, *p* = 0.424), or exits (*β* = 0.08, *p* = 0.081) used.

### Social learning and innovation following demonstrations

We next focused on children who elected to observe demonstrations, examining imitation and innovation-by-modification outcomes.

#### Imitation

Imitation counts (M = 19.60, SD = 6.60) were analysed using a NB2 model with an OLRE. There was no evidence that sociometric indices predicted imitative behaviour (out-degree: *β* = −0.04, SE = 0.02, *z* = −1.75, *p* = 0.081; reciprocity: *β* = 0.04, SE = 0.04, *z* = 0.94, *p* = 0.341; in-degree: *β* = −0.02, SE = 0.02, *z* = −0.71, *p* = 0.480). Age was also not a significant predictor (*β* = −0.04, SE = 0.03, *z* = −1.68, *p* = 0.094).

#### Innovation-by-modification

We analysed three forms of innovation-by-modification following demonstrations: exit innovations (M = 0.83, SD = 1.96), tool innovations (M = 4.24, SD = 3.27), and entrance innovations (M = 2.52, SD = 2.58). Exit and entrance innovations were modelled using NB2 models with OLREs; tool innovations required a ZI-NB2 model with an OLRE. Across all innovation outcomes, sociometric indices did not predict innovative behaviour (exit innovations: all *p*s ≥ 0.187; tool innovations: all *p*s ≥ 0.349; entrance innovations: all *p*s ≥ 0.415).

In contrast, age emerged as a consistent predictor of innovation-by-modification. Older children produced more exit innovations (*β* = 0.61, SE = 0.22, 95% CI [0.18, 1.04], *z* = 2.81, *p* = 0.005) and more entrance innovations (*β* = 0.15, SE = 0.08, 95% CI [0.003, 0.30], *z* = 2.00, *p* = 0.046) (see Figure 4). Age did not predict tool innovations (*β* = 0.04, SE = 0.04, *z* = 0.81, *p* = 0.414).

**Figure 4:**
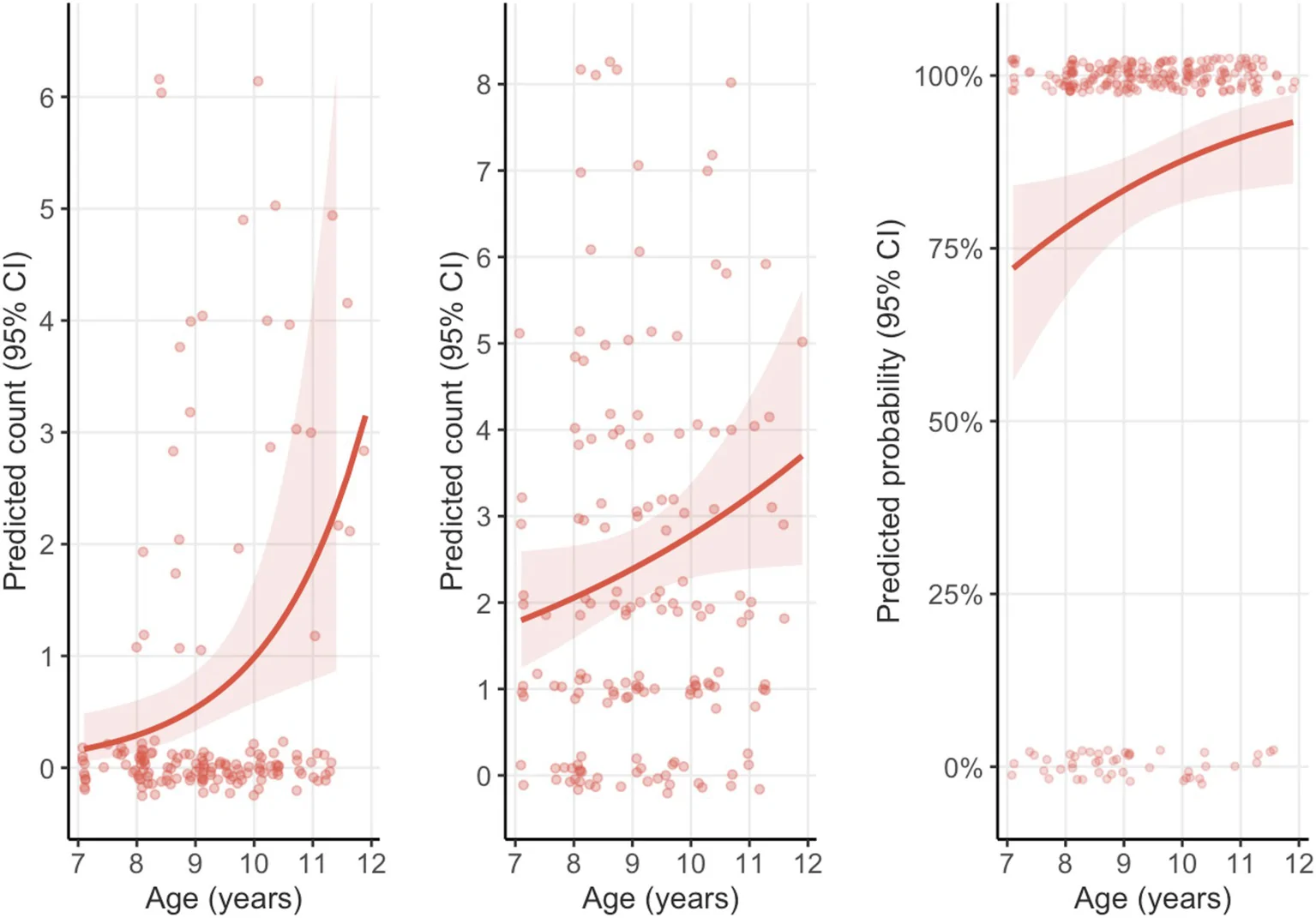
The association between age and a MMB exit innovations (left), MMB entrance innovation (middle), and hook task success (right).

### Asocial exploration and repetition

For the 93 children who elected to solve the MMB without demonstrations, we examined exploratory breadth (number of tools: M = 2.63, SD = 0.99; entrances: M = 3.06, SD = 1.11; exits: M = 2.09, SD = 0.95) and number of method repetitions (M = 3.67, SD = 2.35). Exploration measures were analysed using ZI-NB2 models with OLREs; DHARMa diagnostics flagged residual overdispersion in all three exploration models (all *p*s < 0.001). The tools model additionally produced a non-positive-definite Hessian at convergence and thus these results should be treated with caution. Repetitions were analysed using a NB2 model with an OLRE, which passed all diagnostic checks. There was no evidence that sociometric indices predicted any aspect of asocial exploration or method repetition (tools: all *p*s ≥ 0.546; entrances: all *p*s ≥ 0.501; exits: all *p*s ≥ 0.607; repetitions: all *p*s ≥ 0.183). Age was also not associated with exploration breadth or repetition frequency (all *p*s ≥ 0.546).

### Hook task performance

Overall, 82% of children successfully solved the hook task (*N* = 210), while 18% were unsuccessful (*N* = 46). Among successful participants, the majority bent the pipe cleaner into a hook to retrieve the bucket (*n* = 140, 67%); a substantial minority used a dragging strategy without modifying the tool (*n* = 60, 29%); and a small number employed other improvised methods—including hook-and-string combinations, a pincer or u-shape construction —and were classified into an other category (*n* = 10, 5%). Mean latency to successful retrieval was 44.58 s (SD = 32.69).

There was no evidence that sociometric position predicted hook task success (out-degree: *β* = 0.03, SE = 0.13, *z* = 0.23, *p* = 0.824; reciprocity: *β* = 0.27, SE = 0.33, *z* = 0.82, *p* = 0.412; in-degree: *β* = 0.31, SE = 0.19, *z* = 1.63, *p* = 0.103). Age was positively associated with success (*β* = 0.35, SE = 0.16, *z* = 2.22, *p* = 0.026, OR = 1.42, 95% CI [1.04, 1.93]), with older children more likely to succeed.

To examine whether sociometric indices predicted solution method, we fitted a multinomial logistic regression (with drag as the reference category) among the 210 successful children. There was no evidence that sociometric indices predicted method choice (hook vs. drag—out-degree: *z* = −0.30, *p* = 0.766; reciprocity: *z* = 0.76, *p* = 0.450; in-degree: *z* = −0.81, *p* = 0.419). Age, however, was strongly associated with method: older children were substantially more likely to use a hook than a dragging strategy (*β* = 0.61, SE = 0.16, *z* = 3.94, *p* < 0.001, OR = 1.84, 95% CI [1.36, 2.50]). Age also positively predicted using the other method relative to dragging (*β* = 1.01, SE = 0.33, *z* = 3.01, *p* = 0.003), though estimates for this contrast should be interpreted cautiously given the small other-category sample (*n* = 10). For the effect of age by hook task method type, see Figure 5.

**Figure 5:**
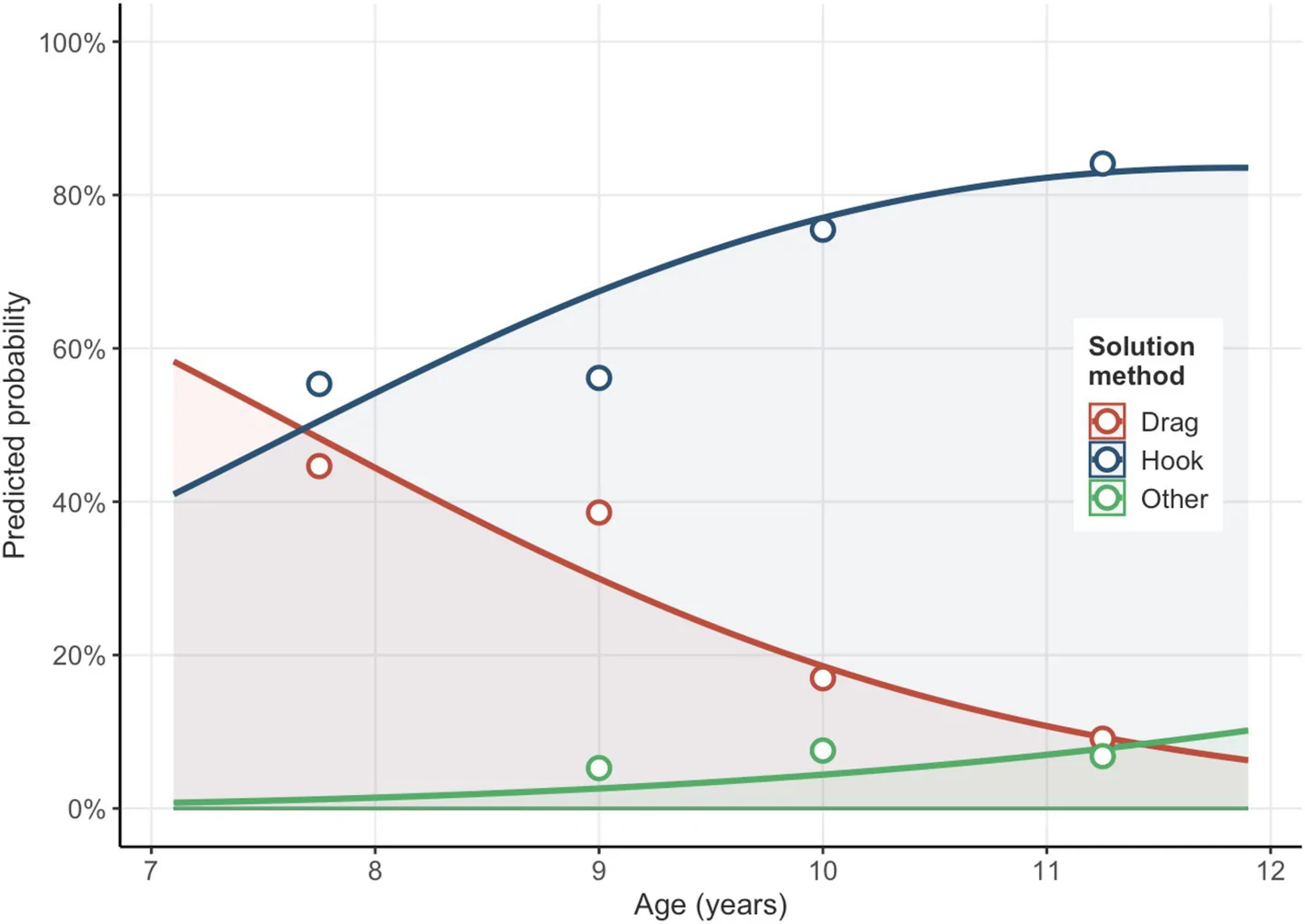
Predicted Hook task methods by age.

Among children who successfully retrieved the reward, a linear mixed-effects model predicting latency from sociometric indices and age revealed no significant effects (out-degree: *β* = 1.34, SE = 1.97, *p* = 0.498; reciprocity: *β* = 1.45, SE = 3.44, *p* = 0.673; in-degree: *β* = 0.28, SE = 1.97, *p* = 0.886; age: *β* = −2.64, SE = 2.27, *p* = 0.254).

### Divergent thinking

Finally, we examined whether sociometric indices predicted divergent thinking on the Alternate Uses task. Mean fluency was 4.89 paperclip uses (SD = 3.05), and mean originality was 3.93 paperclip uses (SD = 5.70; 28.9% of participants scored zero on originality, indicating a substantial floor effect). Sociometric indices were not associated with fluency (out-degree: *β* = 0.02, SE = 0.16, *p* = 0.880; reciprocity: *β* = −0.13, SE = 0.30, *p* = 0.660; in-degree: *β* = −0.06, SE = 0.17, *p* = 0.709) or originality (out-degree: *β* = −0.21, SE = 0.30, *p* = 0.481; reciprocity: *β* = −0.69, SE = 0.56, *p* = 0.216; in-degree: *β* = 0.43, SE = 0.32, *p* = 0.173). Age was a positive predictor of fluency (*β* = 0.38, SE = 0.19, 95% CI [0.01, 0.74], *t*(26.05) = 2.01, *p* = 0.044), indicating that older children generated more uses. Age did not predict originality (*β* = 0.11, SE = 0.35, *t*(22.13) = 0.34, *p* = 0.736). Given that 28.9% of participants scored zero on originality, the LMM residual assumptions are likely strained for this outcome and results for AU originality should be interpreted with caution. For full model details of all models, see the supplementary information.

Age was weakly and negatively associated with out-degree (*r* = −0.21, *p* = 0.001) and in-degree (*r* = −0.13, *p* = 0.035): older children nominated, and were nominated by, slightly fewer classmates. Age was unrelated to reciprocated friendships (*r* = −0.06, *p* = 0.36). Standardising in-degree and reciprocity within classroom (to adjust for classroom size and participation) did not alter the pattern of null sociometric associations reported above (Supplementary Table S11).

## Discussion

Across a battery of puzzlebox, tool innovation, and divergent thinking tasks, we found no evidence that children’s classroom sociometric position shaped how they learned, explored, or solved novel problems. When controlling for age and classroom clustering, none of our three sociometric indices—number of friends nominated (out-degree), number of reciprocated friendships, or number of nominations received from classmates (in-degree)—predicted children’s initial explicit learning strategy choice on the Multi-Method Box, their imitation fidelity, their tendency to innovate by modifying demonstrated methods, their exploratory breadth when solving the MMB asocially, their success or solution method on the hook task, nor their divergent thinking performance on the Alternate Uses task. In contrast, age emerged as a consistent predictor of innovation-related outcomes across tasks, including tool modification, hook task success, and divergent thinking fluency, replicating well-established developmental findings (Beck et al., 2011; Carr et al., 2015; Rawlings and Legare, 2021; Said-Metwaly et al., 2020).

These null findings complement our prior work using the same sample, tasks, and coding scheme, in which stable individual differences in Big Five personality traits predicted children’s learning strategies across the same paradigms (Rawlings et al., 2022). Thus, our results suggest that dispositional and developmental factors may carry greater explanatory weight than peer network embeddedness when accounting for individual variation in social learning and innovation during middle childhood.

### Does network embeddedness matter for children’s learning strategies?

A central aim of the present study was to test whether United Kingdom children’s relational position within their classroom peer ecology predicted their propensity to copy or innovate when facing novel problems. We hypothesized that children with more extensive and more reciprocated classroom friendships may encounter more diverse behavioural models, feel greater normative pressure to conform, or be more attuned to social information more generally (Gest et al., 2003; Haun and Tomasello, 2011; Kratzer and Lettl, 2008; Rawlings et al., 2017). Yet our results do not support these possibilities. Children with more nominated friends, more mutual friendships, or greater peer popularity were no more—or less—likely to elect for social demonstrations, imitate a shown technique, or deviate from observed solutions than children who were less embedded in their classroom networks.

One potential explanation concerns the nature of the experimental context. Children in the present study were deciding whether to receive a social demonstration from an adult experimenter — a choice between relying on social versus asocial information, rather than a choice between specific social sources. This paradigm has considerable value for isolating individual differences in the fundamental decision to seek social information at all, and for examining how that choice relates to subsequent imitation and innovation behaviour. However, sociometric indices of peer embeddedness reflect children’s relational standing within their classroom peer network and may be most consequential in contexts where peer-to-peer social learning is occurring. For instance, when children observe classmates, seek help from friends, or navigate competing peer models. In the present study, the social option was a fixed adult demonstrator whose competence and relevance to the task were independent of children’s peer network position. Children’s decisions about whether to observe that demonstrator may therefore be unlikely to be driven by how many friends they have or how popular they are among classmates. The sociometric indices we used—out-degree, reciprocity, and in-degree—capture social embeddedness within the peer ecology (Cillessen and Marks, 2017; Rubin et al., 2007), but this embeddedness may have limited bearing on children’s willingness to rely on an adult’s social information when facing a novel, individually administered task.

This interpretation aligns with cultural evolutionary theory’s distinction between patterns of interaction—who is connected to whom—and the cognitive and motivational biases that shape how individuals engage with social information (Boyd and Richerson, 1985; Kendal et al., 2018; Mesoudi, 2011). Network structure shapes transmission pathways and population-level diffusion dynamics, but individual learning strategy decisions in experimental contexts may be governed primarily by motivational and developmental processes operating at a different explanatory level (Smolla and Akçay, 2023). It is worth noting, however, that network position might have been more consequential had the paradigm involved choice among social sources rather than the binary social-versus-asocial decision examined here. Highly socially embedded children routinely encounter more social information from more diverse sources, and may consequently develop greater selectivity in evaluating that information—for instance, becoming more attuned to differences in peer competence or demonstrator success. Whether network centrality predicts selective social learning in contexts involving multiple peer models, competing demonstrations, or naturalistic group transmission settings remains an open and important question. We address this further in the Future Directions section.

It is important to consider why we did not employ a peer, rather than an adult, demonstrator. Although a peer model may ostensibly align more closely with the sociometric predictors we studied, it would also introduce potential confounds that would make the results difficult to interpret. Using a specific classmate as the demonstrator would allow that model’s identity, perceived competence, and, crucially, their relationship to the observing child to vary across every participant, confounding network position with the particular dyadic tie between learner and model. Isolating these influences would require a fully dyadic design rather than the individually administered paradigm employed here. Using a single standardised peer, by contrast (for instance, an unfamiliar child or a filmed child demonstrator) would hold the model constant, but that demonstrator would be a stranger to every participant, such that a child’s own sociometric position would again hold no relational connection to the model. Here, sociometric properties would be predicting a decision about a demonstrator to whom the child has no friendship tie, which is the context we used with an adult demonstrator. Viewed this way, the adult demonstrator provides one reasonable operationalisation of the question we set out to test: whether a child’s general orientation toward social information, as opposed to their relationship with any particular model, is shaped by their embeddedness within the classroom peer group. However, we acknowledge that our study cannot address the distinct, relational possibility that embeddedness governs learning specifically from close or familiar peers, since testing that account requires children to choose among identifiable social sources that differ in their network relationship to the learner. Such a design, in which children select which peer to observe, would provide the appropriate test of relational effects and represents an important direction for future work.

### Dispositional versus structural predictors

A further possibility is that the absence of network effects reflects overlap with the personality traits that did predict learning strategies in this sample (Rawlings et al., 2022); if network position and personality captured the same variance, network nulls would be unsurprising. We examined this directly. In that study, personality traits were strongly associated with learning strategies in the same sample and tasks (Rawlings et al., 2022). In that study, agreeableness predicted electing for demonstrations on the MMB, conscientiousness predicted going it alone, and openness to experience predicted innovation-by-modification. However, these personality profiles do not map straightforwardly onto network centrality. For instance, agreeable children are not necessarily the most connected, nor are conscientious children the most peripheral (or vice versa). To examine this directly, we computed pairwise Pearson correlations between each of the Big Five personality traits reported in Rawlings et al. (2022) and each sociometric index in the present sample (*N* = 256). The results confirmed that personality traits and network position were largely independent. The largest associations were between agreeableness and in-degree (*r* = 0.22) and agreeableness and reciprocated friends (*r* = 0.21), theoretically coherent but modest relationships reflecting the tendency for agreeable children to be nominated as friends by more classmates and have more reciprocated friendships. All remaining correlations were small in magnitude (*r* ≤ 0.12; see Supplementary Table S10). These results indicate that the null network findings reported here cannot be attributed to shared variance with the personality traits known to predict learning strategies in this sample. Rather, dispositional orientations and structural network position appear to capture meaningfully distinct aspects of children’s social lives, with the former carrying substantially more explanatory weight for individual learning strategy decisions in experimental contexts.

This dissociation suggests that dispositional orientations toward social information may be more directly relevant to strategy decisions than structural network position, because they reflect motivational and affective states that are activated in the moment of choice. Agreeableness encompasses the propensity to be prosocial, cooperative, and trusting, characteristics that make an individual receptive to social demonstrations, potentially irrespective of their network position (Freitag and Bauer, 2015; Mooradian et al., 2006). Openness to experience reflects a general orientation toward exploration and curiosity that persists across contexts (Abu Raya et al., 2023; Church et al., 2008). Network position, by contrast, may be a more distal background variable, capturing the structure of children’s social environment rather than the motivational or cognitive processes directly determining their learning strategies.

### The role of age on learning strategies

Age consistently predicted innovation across all tasks. Middle childhood is characterised by substantial growth in executive function and metacognition (Best and Miller, 2010; Diamond, 2013)-cognitive processes that support innovation (Rawlings and Legare, 2021), flexible problem-solving (Davis et al., 2022) and social information use (Wilks et al., 2021). Importantly, executive function gains are linked to improvements in social cognition, including theory of mind and perspective-taking, which continue to develop throughout the primary school years (Carlson et al., 2014; Smogorzewska et al., 2020). To the extent that older children are better equipped to represent and evaluate the behaviour of others, age-related advances in these capacities may independently drive more selective and strategic social learning, irrespective of a child’s position within the peer network. Thus, age-related cognitive development may account for substantial variance in learning strategies, potentially overshadowing smaller contextual influences such as classroom network position.

Network effects may also emerge more strongly at different developmental stages. Adolescence is marked by increased time spent with peers and broader peer networks (Brown and Larson, 2009; Wrzus et al., 2013), as well as heightened status sensitivity and peer orientation (Blakemore and Mills, 2014). Importantly, popular peers are known to exert disproportionate social influence during adolescence (Gradassi et al., 2023), with peer-perceived popularity and closeness being associated with both behavioural conformity and the uptake of group norms (Laursen and Veenstra, 2021) and social information use (Gradassi et al., 2023). This suggests that network centrality may exert stronger behavioural influence during that period than was evident in our younger sample. Future work could assess whether the same patterns hold across adolescence.

### Practical implications, motivation, and task context

That age, rather than sociometric position, was the consistent predictor of innovation-related performance presents practical implications. Children’s capacity to imitate effectively and to generate novel solutions was not shaped by their social standing. Thus, less connected or less central children were neither more nor less innovative than their more socially embedded peers. For educators, this suggests that fostering innovation and flexible problem-solving does not depend on a child’s popularity or degree of social integration, and that less central children should not be assumed to contribute less to creative or problem-solving activities. Instead, because age-related developmental gains in executive function and metacognition are perhaps more consequential than sociometric position, classroom activities that scaffold planning, reflection, flexible strategy use, and evaluation of alternative solutions may be especially valuable when pitched to children’s developmental level (Best and Miller, 2010; Diamond, 2013; Rawlings and Legare, 2021). When structuring collaborative work, the absence of sociometric effects implies that educators can compose groups without assuming that socially peripheral children will show a different propensity to imitate or innovate. Heterogeneous groupings may therefore help harness a broader range of contributions, consistent with evidence that peer collaboration can support problem-solving and perspective-taking (Rogoff et al., 2003; Stengelin et al., 2020). We note, however, that our tasks were administered individually, so implications for group-level classroom innovation remain to be tested directly.

Recent work has examined whether children’s motivation may underpin their innovative propensities (Cutting and Neilson, 2026). Our design was not intended to distinguish the motivations underlying children’s choices, and the divergent thinking task deliberately avoided explicit instructions to be creative to minimise demand characteristics (Runco et al., 2005). However, this controlled, individually administered context was useful because it reduced immediate peer-evaluative pressures, allowing us to test whether sociometric position predicted innovation and imitation even when children’s behaviour was not directly visible to classmates. Theoretically, innovation and exploration are often linked to intrinsic drives such as curiosity, openness, and the pleasure of discovering effective solutions (Abu Raya et al., 2023; Bonawitz et al., 2011; Evans et al., 2021). One possibility, consistent with the null sociometric findings, is that any external validation available in the task came primarily from the adult testing context rather than from classmates. If children’s behaviour was not visible to their peer group, their position within that group may have been less likely to shape their choices. Disentangling intrinsic satisfaction from a desire for adult or peer approval would require designs that manipulate audience, feedback, and social visibility directly, and represents a useful direction for future work.

Finally, it is important to reflect on how the tasks relate to the social dynamics of everyday innovation. Each task is a well-established experimental paradigm that indexes a different target construct. Administering all three therefore provided a broad assessment of children’s innovative and imitative competencies, and allowed us to test whether the null sociometric effects generalised across learning-strategy choice, tool innovation, and divergent thinking. Yet, as noted, all three tasks were administered individually, and the social-learning component involved an adult rather than a peer model. The same features that afford experimental control, including a standardised demonstrator, individual testing, and clearly defined outcome measures, also reduce the peer-populated, socially visible context in which sociometric position may show greater influence. Children’s real-world innovation often unfolds in group settings where ideas, successes, and behavioural variants can be observed, evaluated, and transmitted horizontally. The present findings therefore identify an important boundary condition: sociometric position did not predict innovation or imitation in individually administered, experimentally controlled tasks. A useful next step will be to test whether different patterns emerge in more socially visible, group-based paradigms, such as peer-choice designs, collaborative problem-solving tasks, or classroom transmission-chain studies.

### Future directions

Our findings outline important boundary conditions for the role of peer network structure in children’s learning strategies. However, they also open several potentially productive avenues for future research.

The sociometric indices used here reflect children’s relational standing within their peer group, but in the experimental context children were deciding whether to rely on an adult experimenter’s demonstration rather than selecting from among network peers. The factors known to govern model-choice—demonstrator competence, prestige, success rate, and domain relevance—are orthogonal to how many friends a child has. Further, cultural evolutionary theory distinguishes between patterns of interaction (who is connected to whom) and cues used to evaluate potential models (e.g., prestige, competence, success, age; Kendal et al., 2018). Future research should further investigate network metrics with, for example, measures of perceived competence, domain-specific expertise, and status hierarchies, as has been done with capuchin monkeys (Coelho et al., 2024). For example, children may not copy popular peers but may preferentially copy those perceived as skilled in problem-solving tasks. Incorporating peer-rated competence or experimentally manipulated prestige cues would allow more precise tests of how network position and model-based biases jointly shape explicit learning strategy use over development (Wood et al., 2015).

Network embeddedness may also prove more consequential when children choose *among* social sources rather than between social and asocial routes, as in the present paradigm. Future work could examine whether centrality predicts selectivity among social options. For instance, preferential copying of high-status or competent peers, or sensitivity to majority consensus in tasks presenting multiple demonstrators simultaneously. Only a design in which children choose among identifiable peers who differ in their network relationship to the learner can isolate relational effects from the general social orientation examined here. Reciprocated friendships could also be used to test whether friends resemble one another in their learning strategies (homophily), although this requires modelling similarity within friend dyads and, ideally, a measure of learning directed at specific network partners rather than a standardised adult.

Our tasks were administered individually and did not involve open peer diffusion contexts (Mesoudi and Whiten, 2008). Network effects may emerge more strongly in socially visible settings where children observe one another’s success and behavioural variants can spread horizontally (Dean et al., 2012; McGuigan et al., 2017). Implementing learning strategy tasks within group-based transmission chains would allow examination of whether network centrality predicts variant diffusion, modification, or retention across children (Flynn and Whiten, 2010). Such designs would also more closely approximate the natural dynamics of cumulative culture.

Network measures could be extended beyond degree and reciprocity to include structural diversity and brokerage positions. Relatedly, although the fixed five-nomination limit used here is standard and closely matches the average number of friends children nominate at this age (Neal, 2025), free-choice (unlimited) nomination procedures would capture the full upper tail of out-degree and allow this boundary condition to be tested directly. Further, children occupying bridging roles between otherwise disconnected peers may be exposed to more diverse information, potentially increasing opportunities for recombination and innovation (Kratzer and Lettl, 2008). Integrating bridging metrics (Burt, 2004), or measures of network modularity may therefore reveal associations potentially obscured by simpler degree-based indices.

Finally, we encourage developmental extensions to older age groups. As peer networks expand in size and status differentiation intensifies across adolescence (Brown and Larson, 2009; Wrzus et al., 2013), centrality may become more consequential for learning strategies. Longitudinal designs spanning middle childhood into adolescence would help determine whether the relative influence of dispositional traits and network position shifts across development.

## Conclusion

Cumulative culture depends on the interplay between innovation and social learning, yet the sources of individual variation in these processes remain incompletely understood. The present study extends developmental cultural evolution research by testing whether children’s classroom sociometric position predicts their propensity to copy or innovate across multiple tasks. In contrast to dispositional (personality) predictors identified in previous work, sociometric indices of network embeddedness were not associated with learning strategy choice, imitation fidelity, tool innovation, or divergent thinking.

These findings refine our understanding of the “social” in social learning. Relational embeddedness within a peer network did not determine whether children exploited social information or engaged in exploration when facing novel problems. Rather, learning strategy decisions may be instead shaped by metacognitive evaluation, motivational orientation, and model-based cues. Network structure may shape opportunities for interaction and diffusion, but the selective uptake and modification of information appears to operate at a different explanatory level. These findings suggest that the ontogeny of cumulative culture may involve shifting contributions from individual dispositions, model-based biases, and network structure across development. Understanding how these components interact will be essential for explaining how new behavioural variants emerge, spread, and stabilize within populations.

By integrating sociometric methods with experimental measures of imitation and innovation, our study lays groundwork for mechanistic accounts of how children’s cultural traditions are generated and transmitted. Future work bridging network science, developmental research, and cultural evolution theory will be critical for advancing this line of research.

## Data Availability

The datasets presented in this study can be found in online repositories. The names of the repository/repositories and accession number(s) can be found below: 10.6084/m9.figshare.31851571.
