# Supplementary material for: It’s not who you know: sociometric position did not shape children’s innovation or imitation propensities
Source: Front Psychol. 2026 Oct 16;17:1838722. doi: 10.3389/fpsyg.2026.1838722 (PMC13719602; doi:10.3389/fpsyg.2026.1838722)
Supplement: Supplementary file 1 [file Supplementary_file_1.pdf]

Supplementary Materials

It's not who you know: friendship networks did not shape children's innovation or imitation propensities

Bruce Rawlings, Emma Flynn, Rachel Kendal

Contents: S1. VIF table | S2. MMB learning strategy choice | S3. MMB all participants | S4. MMB demonstrations subset | S5. MMB asocial subset | S6. Hook task | S7. Alternate Uses task | S8. DHARMa diagnostics | S9. LMM residual diagnostics | S10. Personality–sociometric correlations

S1. Multicollinearity: Variance Inflation Factors

Prior to model fitting, variance inflation factors (VIFs) were computed using a standard linear regression of AU fluency on all predictors (outcome choice does not affect VIF values, which depend only on the predictor matrix). All VIFs were below the threshold of concern (VIF > 5), indicating no problematic collinearity among predictors.

Predictor	VIF	Interpretation
Number of friends nominated (out-degree)	1.12	No concern
Reciprocated friendships	2.51	No concern
Times nominated by peers (in-degree)	2.49	No concern
Age	1.06	No concern

S2. MMB Learning Strategy Choice (Binomial GLMM)

Model: Binomial mixed-effects logistic regression (glmer, bobyqa optimiser). Outcome: MMB learning strategy choice (0 = elected for social demonstration, 1 = no demonstration). Random intercept: classroom (school × year group). Note: classroom random-effect variance was estimated at zero (singular fit); a sensitivity analysis using a plain logistic GLM produced identical parameter estimates. N = 256.

Note on effect sizes: β = log-odds coefficient. OR = odds ratio = exp(β). 95% CI on OR scale in brackets.

Predictor	β	SE	z	p	OR	95% CI
Intercept	−1.21	1.04	−1.17	.244	—	—
Out-degree	−0.09	0.10	−0.84	.400	0.92	[0.75, 1.12]
Reciprocated friendships	0.04	0.21	0.19	.853	1.04	[0.69, 1.56]
In-degree	−0.06	0.12	−0.48	.629	0.94	[0.75, 1.20]
Age	0.20	0.11	1.76	.079	1.22	[0.98, 1.53]

Bold rows indicate p < .05. Classroom random-effect variance = 0 (singular). Sensitivity GLM: identical estimates. Singular fit reported in main text.

S3. MMB Behavioural Outcomes — All Participants

All models: glmmTMB with classroom random intercept and observation-level random effect (OLRE). NB2 = negative binomial; ZI-NB2 = zero-inflated NB2. N = 256.

S3a. Failed Attempts (NB2 + OLRE)

DHARMa: all checks passed. β = log count coefficient; IRR = exp(β).

Predictor	β	SE	z	p	IRR	95% CI
**Intercept**	**2.44**	**0.52**	**4.69**	**< .001**	**—**	**—**
Out-degree	0.04	0.04	0.93	.345	1.04	[0.96, 1.12]
Reciprocated friendships	−0.10	0.07	−1.45	.150	0.91	[0.80, 1.04]
In-degree	−0.02	0.04	−0.62	.545	0.98	[0.91, 1.05]
Age	−0.05	0.04	−1.11	.262	0.95	[0.88, 1.03]

S3b. Number of Different Tools Used — All Participants (ZI-NB2 + OLRE)
DHARMa: residual overdispersion flagged (p < .001). Results should be interpreted with caution.

Predictor	β	SE	z	p	IRR	95% CI
**Intercept**	**0.93**	**0.38**	**2.47**	**.014**	**—**	**—**
Out-degree	0.03	0.03	0.95	.340	1.03	[0.97, 1.10]
Reciprocated friendships	0.03	0.06	0.50	.615	1.03	[0.91, 1.17]
In-degree	−0.02	0.04	−0.51	.611	0.98	[0.91, 1.06]
Age	−0.001	0.04	−0.02	.983	1.00	[0.93, 1.07]

S3c. Number of Different Entrances Used — All Participants (ZI-NB2 + OLRE)
DHARMa: residual overdispersion flagged (p < .001). Results should be interpreted with caution.

Predictor	β	SE	z	p	IRR	95% CI
**Intercept**	**1.12**	**0.40**	**2.80**	**.005**	**—**	**—**
Out-degree	0.02	0.03	0.69	.491	1.02	[0.96, 1.09]
Reciprocated friendships	−0.04	0.06	−0.60	.547	0.96	[0.85, 1.09]
In-degree	−0.01	0.04	−0.15	.880	0.99	[0.92, 1.07]
Age	0.03	0.03	0.80	.424	1.03	[0.96, 1.10]

S3d. Number of Different Exits Used — All Participants (ZI-NB2 + OLRE)
DHARMa: residual overdispersion flagged (p < .001). Results should be interpreted with caution.

Predictor	β	SE	z	p	IRR	95% CI
Intercept	0.60	0.38	1.59	.112	—	—
Out-degree	−0.01	0.04	−0.23	.818	0.99	[0.92, 1.07]
Reciprocated friendships	−0.04	0.08	−0.56	.573	0.96	[0.82, 1.12]
In-degree	0.02	0.04	0.51	.608	1.02	[0.94, 1.11]
Age	0.08	0.04	1.74	.081	1.08	[1.00, 1.17]

S4. MMB — Social Learning and Innovation Following Demonstrations
Subset: children who elected to observe demonstrations (N = 163). All models: glmmTMB with classroom random intercept and OLRE.

S4a. Imitation Score (NB2 + OLRE)
DHARMa: all checks passed. M = 19.60, SD = 6.60.

Predictor	β	SE	z	p	IRR	95% CI
**Intercept**	**3.47**	**0.29**	**11.84**	**< .001**	**—**	**—**
Out-degree	−0.04	0.02	−1.75	.081	0.96	[0.91, 1.00]
Reciprocated friendships	0.04	0.04	0.94	.341	1.04	[0.96, 1.12]
In-degree	−0.02	0.02	−0.71	.480	0.98	[0.94, 1.02]
Age	−0.04	0.03	−1.68	.094	0.96	[0.92, 1.01]

S4b. Exit Innovations (NB2 + OLRE)
DHARMa: all checks passed. M = 0.83, SD = 1.96.

Predictor	β	SE	z	p	IRR	95% CI
Intercept	−4.25	2.22	−1.92	.055	—	—
Out-degree	0.28	0.21	1.33	.187	1.33	[0.87, 2.02]
Reciprocated friendships	−0.51	0.42	−1.21	.226	0.60	[0.27, 1.37]
In-degree	0.15	0.22	0.66	.507	1.16	[0.75, 1.79]
**Age**	**0.61**	**0.22**	**2.81**	**.005**	**1.84**	**[1.20, 2.83]**

S4c. Tool Innovations (ZI-NB2 + OLRE)
DHARMa: all checks passed. M = 4.24, SD = 3.27. ZI model required due to excess zeros.

Predictor	β	SE	z	p	IRR	95% CI
**Intercept**	**1.71**	**0.39**	**4.38**	**< .001**	**—**	**—**
Out-degree	0.04	0.05	0.73	.467	1.04	[0.94, 1.14]
Reciprocated friendships	−0.01	0.07	−0.10	.920	0.99	[0.86, 1.14]
In-degree	0.04	0.04	0.94	.349	1.04	[0.96, 1.12]
Age	0.04	0.04	0.81	.414	1.04	[0.95, 1.13]

S4d. Entrance Innovations (NB2 + OLRE)
DHARMa: all checks passed. M = 2.52, SD = 2.58.

Predictor	β	SE	z	p	IRR	95% CI
Intercept	−0.26	0.81	−0.32	.750	—	—
Out-degree	0.04	0.07	0.56	.572	1.04	[0.91, 1.19]
Reciprocated friendships	−0.10	0.12	−0.82	.415	0.91	[0.72, 1.15]
In-degree	0.05	0.07	0.66	.509	1.05	[0.91, 1.20]
**Age**	**0.15**	**0.08**	**2.00**	**.046**	**1.16**	**[1.003, 1.35]**

S5. MMB — Asocial Exploration (No-Demonstration Subset)

Subset: children who attempted the MMB without demonstrations (N = 93). All models: glmmTMB with classroom random intercept and OLRE.

S5a. Number of Different Tools — Asocial (ZI-NB2 + OLRE)

DHARMa: residual overdispersion flagged (p < .001). Additionally, non-positive-definite Hessian at convergence (potential over-parameterisation in this subsample). Results should be treated with considerable caution.

Predictor	β	SE	z	p	IRR	95% CI
Intercept	0.85	0.62	1.38	.168	—	—
Out-degree	0.02	0.05	0.39	.696	1.02	[0.92, 1.13]
Reciprocated friendships	−0.01	0.10	−0.10	.918	0.99	[0.81, 1.21]
In-degree	−0.01	0.06	−0.09	.928	0.99	[0.88, 1.12]
Age	−0.03	0.06	−0.60	.546	0.97	[0.86, 1.09]

S5b. Number of Different Entrances — Asocial (ZI-NB2 + OLRE)

DHARMa: residual overdispersion flagged (p < .001). Results should be interpreted with caution.

Predictor	β	SE	z	p	IRR	95% CI
Intercept	1.09	0.63	1.72	.086	—	—
Out-degree	0.01	0.04	0.31	.759	1.01	[0.93, 1.10]
Reciprocated friendships	0.01	0.10	0.11	.908	1.01	[0.83, 1.24]
In-degree	−0.04	0.06	−0.67	.501	0.96	[0.86, 1.08]
Age	−0.02	0.05	−0.33	.742	0.98	[0.89, 1.08]

S5c. Number of Different Exits — Asocial (ZI-NB2 + OLRE)

DHARMa: residual overdispersion flagged (p < .001). Results should be interpreted with caution.

Predictor	β	SE	z	p	IRR	95% CI
Intercept	0.76	0.65	1.17	.241	—	—
Out-degree	−0.02	0.05	−0.43	.666	0.98	[0.88, 1.08]
Reciprocated friendships	0.002	0.11	0.02	.987	1.00	[0.81, 1.24]
In-degree	0.03	0.06	0.52	.607	1.03	[0.91, 1.17]
Age	−0.01	0.06	−0.18	.857	0.99	[0.87, 1.12]

S5d. Method Repetitions — Asocial (NB2 + OLRE)

DHARMa: all checks passed. M = 3.67, SD = 2.35.

Predictor	β	SE	z	p	IRR	95% CI
**Intercept**	**1.63**	**0.67**	**2.43**	**.015**	**—**	**—**
Out-degree	−0.07	0.05	−1.33	.183	0.94	[0.85, 1.03]
Reciprocated friendships	0.13	0.10	1.24	.217	1.14	[0.93, 1.38]

In-degree	0.003	0.06	0.05	.960	1.00	[0.89, 1.13]
Age	0.03	0.06	0.47	.624	1.03	[0.91, 1.16]

S6. Hook Task Models

S6a. Hook Task Success (Binomial GLMM)

Model: glmer (bobyqa). Outcome: success (1) vs failure (0). Random intercept: classroom. N = 256. DHARMa: passed.

Predictor	β	SE	z	p	OR	95% CI
Intercept	−1.68	1.52	−1.11	.268	—	—
Out-degree	0.03	0.13	0.23	.824	1.03	[0.80, 1.33]
Reciprocated friendships	0.27	0.33	0.82	.412	1.31	[0.69, 2.49]
In-degree	0.31	0.19	1.63	.103	1.37	[0.94, 1.98]
**Age**	**0.35**	**0.16**	**2.22**	**.026**	**1.42**	**[1.04, 1.93]**

S6b. Hook Task Solution Method — Multinomial Logistic Regression

Subset: successful participants only (N = 210). Reference category: drag. Method categories: hook (n = 140, 66.7%), drag (n = 60, 28.6%), other (n = 10, 4.8%). Note: no classroom random effect (nnet::multinom does not support random effects). Interpret cautiously — classroom clustering uncontrolled. Estimates for the "other" contrast are particularly uncertain given the small cell (n = 10).

Hook vs Drag contrast:

Predictor	β	SE	z	p	OR	95% CI
**Intercept**	**−4.10**	**1.62**	**−2.54**	**.011**	**—**	**—**
Out-degree	−0.05	0.16	−0.30	.766	0.96	[0.70, 1.31]
Reciprocated friendships	0.27	0.36	0.76	.450	1.31	[0.65, 2.65]
In-degree	−0.15	0.19	−0.81	.419	0.86	[0.59, 1.25]
**Age**	**0.61**	**0.16**	**3.94**	**< .001**	**1.84**	**[1.36, 2.50]**

Other vs Drag contrast (n = 10; interpret with caution):

Predictor	β	SE	z	p	OR	95% CI
**Intercept**	**−11.13**	**3.33**	**−3.34**	**.001**	**—**	**—**
Out-degree	0.15	0.38	0.40	.693	1.16	[0.55, 2.46]
Reciprocated friendships	0.06	0.74	0.09	.931	1.07	[0.25, 4.55]
In-degree	−0.13	0.41	−0.31	.753	0.88	[0.39, 1.97]
**Age**	**1.01**	**0.33**	**3.01**	**.003**	**2.74**	**[1.42, 5.28]**

S6c. Hook Task Latency — Successful Trials Only (LMM)

Subset: N = 210 children who successfully retrieved the reward. Random intercept: classroom. M latency = 44.58 s, SD = 32.69. Residual Q–Q and fitted-vs-residual plots inspected; no gross violations noted.

Predictor	β	SE	t	p	95% CI
**Intercept**	**62.28**	**24.69**	**2.52**	**.016**	**[13.89, 110.67]**
Out-degree	1.34	1.97	0.68	.498	[−2.52, 5.20]
Reciprocated friendships	1.45	3.44	0.42	.673	[−5.29, 8.19]
In-degree	0.28	1.97	0.14	.886	[−3.57, 4.13]
Age	−2.64	2.27	−1.16	.254	[−7.09, 1.81]

S7. Alternate Uses Task (Divergent Thinking)

N = 256. Both models: lmer with classroom random intercept, Satterthwaite approximation for denominator degrees of freedom. Residual diagnostics inspected via Q–Q and fitted-vs-residual plots.

S7a. AU Fluency (LMM)

Residual diagnostics: acceptable. M = 4.89, SD = 3.05.

Predictor	β	SE	t	df	p	95% CI
Intercept	−0.91	1.78	−0.51	27.4	.614	[−4.57, 2.75]
Out-degree	0.02	0.16	0.15	243.1	.880	[−0.29, 0.34]
Reciprocated friendships	−0.13	0.30	−0.44	243.2	.660	[−0.71, 0.45]
In-degree	−0.06	0.17	−0.37	244.1	.709	[−0.39, 0.27]
**Age**	**0.38**	**0.19**	**2.01**	**26.05**	**.044**	**[0.01, 0.74]**

S7b. AU Originality (LMM)

Floor effect: 28.9% of participants (n = 74/256) scored zero on originality. The LMM assumption of normally distributed residuals is likely strained by this floor effect. Results for AU originality should be interpreted with caution.

M = 3.93, SD = 5.70.

Predictor	β	SE	t	df	p	95% CI
Intercept	0.11	3.38	0.03	27.2	.974	[−6.79, 7.01]
Out-degree	−0.21	0.30	−0.71	241.8	.481	[−0.79, 0.38]
Reciprocated friendships	−0.69	0.56	−1.24	241.9	.216	[−1.78, 0.41]
In-degree	0.43	0.32	1.37	242.8	.173	[−0.19, 1.06]
Age	0.11	0.35	0.34	22.13	.736	[−0.57, 0.79]

S8. DHARMa Diagnostic Summary (Count Models and GLMMs)
DHARMa simulated residuals (n = 1000 simulations) were used to assess uniformity, dispersion, zero-inflation, and outliers for all glmmTMB and glmer count models. Linear mixed models (hook latency, AU fluency, AU originality) were assessed by visual inspection of Q–Q and residuals-versus-fitted plots. Multinomial models are excluded from DHARMa. Key: ✓ = passed; = flagged.

Model	Family	Uniformity	Dispersion	Zero-inflation	Outliers	Overall
MMB strategy choice	Binomial GLMM	✓	✓	n/a	✓	✓ Passed
MMB fails (all)	NB2 + OLRE	✓	✓	✓	✓	✓ Passed
MMB tools (all)	ZI-NB2 + OLRE	✓	p<.001	n/a (ZI)	✓	Caution
MMB entrances (all)	ZI-NB2 + OLRE	✓	p<.001	n/a (ZI)	✓	Caution
MMB exits (all)	ZI-NB2 + OLRE	✓	p<.001	n/a (ZI)	✓	Caution
Imitation (demo)	NB2 + OLRE	✓	✓	✓	✓	✓ Passed
Exit innovations (demo)	NB2 + OLRE	✓	✓	✓	✓	✓ Passed
Tool innovations (demo)	ZI-NB2 + OLRE	✓	✓	n/a (ZI)	✓	✓ Passed
Entrance innovations (demo)	NB2 + OLRE	✓	✓	✓	✓	✓ Passed
Tools (asocial)	ZI-NB2 + OLRE	✓	p<.001	n/a (ZI)	✓	Caution + Hessian
Entrances (asocial)	ZI-NB2 + OLRE	✓	p<.001	n/a (ZI)	✓	Caution
Exits (asocial)	ZI-NB2 + OLRE	✓	p<.001	n/a (ZI)	✓	Caution
Repetitions (asocial)	NB2 + OLRE	✓	✓	✓	✓	✓ Passed
Hook success	Binomial GLMM	✓	✓	n/a	✓	✓ Passed
Hook latency (LMM)	Gaussian LMM	—	—	—	—	✓ Visual OK
AU fluency (LMM)	Gaussian LMM	—	—	—	—	✓ Visual OK
AU originality (LMM)	Gaussian LMM	—	—	—	—	Floor effect

Note on ZI-NB2 dispersion flags: Residual overdispersion in ZI-NB2 models suggests that even zero-inflation + OLRE did not fully absorb extra-Poisson variance in these outcomes. This is most likely attributable to the small sample size and high structural heterogeneity in children's exploratory behaviour. All substantive estimates (sociometric predictors) were null, limiting interpretive impact, but these results should be treated as exploratory.

S9. Linear Mixed Model Residual Diagnostics

Three outcomes were analysed using linear mixed-effects models: hook task latency (N = 210), AU fluency (N = 256), and AU originality (N = 256). Residual diagnostics (Q–Q plots and residuals vs. fitted values) were inspected visually.

Hook task latency: Residuals showed mild positive skew consistent with a latency distribution. No severe violations. LMM results are reported without transformation as effects of interest were null.

AU fluency: Residuals were approximately normally distributed with some right skew at higher fluency scores. No severe violations.

AU originality: 28.9% of participants scored zero on originality (74/256), creating a pronounced floor effect. The LMM residuals deviated from normality, particularly at the lower tail. A zero-inflated or hurdle model would be more appropriate but was not fitted to maintain consistency with the fluency model. AU originality results should be interpreted with caution, and this limitation is acknowledged in the manuscript.

S10. Personality–Sociometric Correlations (N = 256)

To examine whether personality traits (reported in Rawlings et al., 2022, same sample) were confounded with sociometric indices, we computed Pearson correlations between the Big Five personality factors and each sociometric index across the full sample (N = 256). All 256 participants had complete data on both personality and sociometric variables. These data were not used in the primary analyses; the personality and network predictors were each tested in separate studies. The purpose here is simply to assess independence.

Personality trait	Out-degree (r)	Reciprocated friends (r)	In-degree (r)
Agreeableness	+.036	+.206	+.219
Conscientiousness	−.037	+.102	+.085
Extraversion	+.123	+.066	+.058
Openness to experience	+.098	+.008	+.047
Neuroticism	−.036	−.089	−.062

The largest correlation was between agreeableness and in-degree ($r = .219$), indicating that children rated as more agreeable tended to be named as a friend by more classmates. All other correlations were small ($|r| \leq .21$). None reached a magnitude that would suggest substantial confounding between dispositional and structural predictors. The null network findings in the present study therefore cannot be attributed to overlap with personality traits known to predict learning strategies in this sample.

Note on interpretation of agreeableness × in-degree: The modest positive association between agreeableness and in-degree as well as reciprocated friendships is theoretically plausible (agreeable children may be preferred as friends) but does not constitute confounding in the analytic sense, as personality was not a covariate in the current models and the two sets of predictors were tested in separate analyses targeting different explanatory questions.

Table S11
