# Supplementary material for: It’s not who you know: sociometric position did not shape children’s innovation or imitation propensities
Source: Front Psychol. 2026 Oct 16;17:1838722. doi: 10.3389/fpsyg.2026.1838722 (PMC13719602; doi:10.3389/fpsyg.2026.1838722)
Supplement: Supplementary file 2 [file Supplementary_file_2.pdf]

Supplementary Table S11. Robustness of associations between sociometric position and behavioural outcomes to within-classroom standardisation. Each cell reports the estimated model coefficient and associated p-value for the sociometric predictor from the same models reported in the main text: glmmTMB NB2/ZI-NB2 models with observation-level random effects, binomial GLMMs, multinomial logistic regression for hook method, and linear mixed-effects models for hook latency and Alternate Uses. All models controlled for age and classroom. Columns present sociometric indices coded as originally analysed (Raw), standardised as a proportion of participating classmates within classroom (Proportional), and standardised as within-classroom z-scores. For the multinomial hook-method analysis, the minimum p-value across contrasts is reported. $p < .05$.

Raw : Out-degree	Raw : In-degree	Raw : Reciprocity	Proportional : Out-degree	Proportional : In-degree	Proportional : Reciprocity	Within-class z : Out-degree	Within-class z : In-degree
0.024 (0.880)	-0.063 (0.709)	-0.130 (0.660)	0.032 (0.837)	-1.308 (0.483)	-1.015 (0.749)	0.056 (0.797)	-0.164 (0.518)
-0.207 (0.481)	0.433 (0.173)	-0.688 (0.216)	-0.233 (0.432)	3.034 (0.390)	-4.271 (0.476)	-0.712 (0.084)	0.538 (0.266)
0.041 (0.572)	0.046 (0.509)	-0.100 (0.415)	0.041 (0.585)	0.636 (0.399)	-1.369 (0.291)	0.286 (0.016)*	0.023 (0.818)
0.022 (0.491)	-0.005 (0.880)	-0.038 (0.547)	0.029 (0.372)	0.062 (0.874)	-0.958 (0.162)	0.039 (0.403)	-0.021 (0.701)
0.013 (0.759)	-0.038 (0.501)	0.011 (0.908)	0.018 (0.691)	-0.046 (0.947)	-0.812 (0.486)	0.029 (0.629)	-0.056 (0.549)
0.283 (0.187)	0.147 (0.507)	-0.509 (0.226)	0.314 (0.279)	2.946 (0.349)	-6.869 (0.230)	0.874 (0.122)	0.376 (0.374)
-0.009 (0.818)	0.022 (0.608)	-0.044 (0.573)	0.005 (0.901)	0.018 (0.970)	-0.905 (0.287)	-0.006 (0.909)	0.020 (0.766)
-0.023 (0.666)	0.034 (0.607)	0.002 (0.987)	0.004 (0.939)	0.082 (0.921)	-0.915 (0.517)	-0.013 (0.857)	0.052 (0.634)
1.341 (0.498)	0.283 (0.885)	1.454 (0.673)	1.558 (0.427)	-0.795 (0.972)	8.375 (0.825)	2.000 (0.445)	1.719 (0.563)
p = 0.429	p = 0.390	p = 0.422	p = 0.213	p = 0.074	p = 0.338	p = 0.625	p = 0.047*
0.029 (0.824)	0.312 (0.103)	0.273 (0.412)	0.036 (0.800)	3.145 (0.142)	3.237 (0.381)	-0.019 (0.917)	0.741 (0.013)*
-0.041 (0.081)	-0.017 (0.480)	0.039 (0.341)	-0.044 (0.057)	-0.062 (0.804)	0.499 (0.238)	-0.094 (0.004)*	-0.007 (0.839)
-0.085 (0.400)	-0.058 (0.629)	0.038 (0.853)	-0.047 (0.644)	-1.953 (0.171)	0.239 (0.919)	-0.170 (0.255)	-0.211 (0.254)
0.035 (0.345)	-0.024 (0.545)	-0.100 (0.150)	0.054 (0.130)	-0.416 (0.335)	-2.197 (0.004)*	0.073 (0.129)	-0.055 (0.346)
-0.066 (0.183)	0.003 (0.960)	0.127 (0.217)	-0.089 (0.069)	-0.371 (0.637)	3.220 (0.007)*	-0.018 (0.790)	0.037 (0.706)
0.038 (0.468)	0.038 (0.349)	-0.007 (0.920)	0.047 (0.373)	0.133 (0.777)	-0.124 (0.880)	0.079 (0.332)	0.016 (0.800)
0.032 (0.340)	-0.018 (0.611)	0.031 (0.615)	0.036 (0.283)	-0.067 (0.865)	-0.059 (0.930)	-0.006 (0.896)	-0.029 (0.604)

Raw : Out-degree	Raw : In-degree	Raw : Reciprocity	Proportional : Out-degree	Proportional : In-degree	Proportional : Reciprocity	Within-class z : Out-degree	Within-class z : In-degree
0.019 (0.696)	-0.005 (0.927)	-0.011 (0.918)	0.021 (0.673)	0.244 (0.735)	-0.702 (0.569)	-0.020 (0.752)	-0.013 (0.901)

Note. No sociometric coefficient was significant using the raw indices. Under proportional and within-classroom standardisation, a small number of isolated coefficients reached $p < .05$. However, none replicated across both standardisation schemes for the same sociometric index and behavioural outcome. The reciprocity effects were attributable to the smallest classroom (n = 2), where proportional standardisation is inherently unstable. Overall, these robustness analyses provide no evidence for a consistent association between sociometric position and children's behavioural performance.
